# Glucose-6-phosphate dehydrogenase blockade potentiates tyrosine kinase inhibitor effect on breast cancer cells through autophagy perturbation

**DOI:** 10.1186/s13046-019-1164-5

**Published:** 2019-04-12

**Authors:** Luigi Mele, Marcella la Noce, Francesca Paino, Tarik Regad, Sarah Wagner, Davide Liccardo, Gianpaolo Papaccio, Angela Lombardi, Michele Caraglia, Virginia Tirino, Vincenzo Desiderio, Federica Papaccio

**Affiliations:** 1 Department of Experimental Medicine, University of Campania “Luigi Vanvitelli”, Via Luciano Armanni, 5, 80138 Napoli, Naples, Italy; 2 Department of Biomedical, Surgical and Dental Sciences, University of Milan, Milan, Italy; 3 Department Precision Medicine, University of Campania “Luigi Vanvitelli”, 80138 Naples, Italy; 4 The John van Geest Cancer Research Centre, School of Science and Technology, Nottingham Trent University, Clifton Lane, Nottingham, NG11 8NS UK; 5 Molecular Oncology Laboratory, Biogem Scarl, Ariano Irpino, Avellino Italy

**Keywords:** Pentose phosphate pathway, Autophagy, Lapatinib, ER stress, TKI, Breast cancer, Polydatin

## Abstract

**Background:**

Glucose-6-phospate dehydrogenase (G6PD) is the limiting enzyme of the pentose phosphate pathway (PPP) correlated to cancer progression and drug resistance. We previously showed that G6PD inhibition leads to Endoplasmic Reticulum (ER) stress often associated to autophagy deregulation. The latter can be induced by target-based agents such as Lapatinib, an anti-HER2 tyrosine kinase inhibitor (TKI) largely used in breast cancer treatment.

**Methods:**

Here we investigate whether G6PD inhibition causes autophagy alteration, which can potentiate Lapatinib effect on cancer cells. Immunofluorescence and flow cytometry for LC3B and lysosomes tracker were used to study autophagy in cells treated with lapatinib and/or G6PD inhibitors (polydatin). Immunoblots for LC3B and p62 were performed to confirm autophagy flux analyses together with puncta and colocalization studies. We generated a cell line overexpressing G6PD and performed synergism studies on cell growth inhibition induced by Lapatinib and Polydatin using the median effect by Chou-Talay. Synergism studies were additionally validated with apoptosis analysis by annexin V/PI staining in the presence or absence of autophagy blockers.

**Results:**

We found that the inhibition of G6PD induced endoplasmic reticulum stress, which was responsible for the deregulation of autophagy flux. Indeed, G6PD blockade caused a consistent increase of autophagosomes formation independently from mTOR status. Cells engineered to overexpress G6PD became resilient to autophagy and resistant to lapatinib. On the other hand, G6PD inhibition synergistically increased lapatinib-induced cytotoxic effect on cancer cells, while autophagy blockade abolished this effect. Finally, in silico studies showed a significant correlation between G6PD expression and tumour relapse/resistance in patients.

**Conclusions:**

These results point out that autophagy and PPP are crucial players in TKI resistance, and highlight a peculiar vulnerability of breast cancer cells, where impairment of metabolic pathways and autophagy could be used to reinforce TKI efficacy in cancer treatment.

## Background

In recent years, metabolic deregulations have been studied as prognostic factors for tumours and as potential targets for innovative treatments [1–4]. Pentose phosphate pathway (PPP), a cytoplasmic metabolic process parallel to glycolysis, synthesizes the nucleotide precursor ribose-5-phosphate and produces NADPH, the reduced form of NADP^+^ (the nicotinamide adenine dinucleotide phosphate), that is an essential cofactor for the synthesis of lipids and maintenance of redox balance of the cell [5, 6]. NADPH counteracts oxidative stress produced in highly metabolizing cancer cells in form of reactive oxygen species (ROS). Glucose-6-phosphate dehydrogenase (G6PD) is the key enzyme of the PPP, it is often over-expressed in several types of cancer such as breast cancer, oesophageal carcinoma, renal cancer and is correlated to worse prognosis [7–10]. Indeed, some of the main oncogenes and tumour suppressors such as p53 and K-Ras, can directly regulate the enzymes involved in this pathway [6, 11, 12]. Several studies showed that the inhibition of G6PD may result in the development of therapeutic strategies against tumour growth and metastasis [13–15].

Breast cancer is the most common type of cancer in women and is associated with high mortality that is due to tumour aggressiveness and drug resistance. This cancer is classified according to the expression of oestrogen receptor (ERec), progesterone receptor (PR), and/or human epidermal growth factor receptor 2 (HER-2). The expression of these receptors determines the phenotype of the breast cancer and thus defines the therapeutic strategy to follow [16, 17]. Lapatinib, an orally administered small-molecule, is a dual tyrosine kinase inhibitor (TKI) that targets both HER-2 receptor and the epidermal growth factor receptor (EGFR), and which has been widely used for the treatment of breast cancer [18]. This inhibitor induces apoptosis and autophagy in cancer cells. However, resistance to lapatinib treatment is also observed. The resistance can be caused by alterations in autophagosome and autolysosome proteins, suggesting a potential role of autophagy [19–21]. Furthermore, the increase of the antioxidant metabolic pathway has been associated with TKI resistance in cancer. This suggests that the strategies based on the targeting of antioxidant metabolic pathways may improve the efficiency of TKI-based therapy [22]. In this study, we investigated the interplay between G6PD, ER stress and autophagy, and highlighted new possible strategies to improve the effect of TKIs in treatment of breast cancer overcoming drug resistance.

## Materials and methods

### Chemicals, cell culture and in vitro treatment

All chemicals were purchased from Sigma-Aldrich (St. Louis, USA) unless otherwise specified. Trans-polydatin, with a purity grade higher than 99%, was been supplied by Ghimas spa (Casalecchio, Bologna, Italy). Selective inhibitors of IRE1α (4μ8C) and PERK (GSK 2606414) were obtained from Tocris Bioscience (Bristol, United Kingdom). Lapatinib (Tyverb®) was obtained from GSK (Verona, Italy). MCF7 cells were purchased from ATCC. Cells were cultured in DMEM (Gibco, NY, USA) supplemented with 2 mM glutamine, 100 IU/mL penicillin, 100 μg/mL streptomycin (Invitrogen, Carlsbad, CA), and 10% heat-inactivated foetal bovine serum (FBS) (Gibco, NY, USA) at 37 °C in a humidified atmosphere under 5% CO2. All cell lines were kept mycoplasma free, checking was performed every three months.

### Cell viability assay

Cell viability was measured by the colorimetric 3-(4,5-dimethyl-2-thiazolyl)-2,5-diphenyltetrazolium bromide (MTT) assay. Cells were seeded in 96-well plates at a density of 10^4^ cells per well, then they were treated with 100 μL of 1 mg/mL MTT (Sigma) in DMEM medium containing 10% foetal bovine serum for 4 h at 37 °C. The medium was then replaced with 200 μL of DMSO and shaken for 15 min, then absorbance at 540 nm was measured using a microplate ELISA reader with DMSO used as the blank. To quantify the synergistic or antagonist effect of the drugs combinations, CompuSyn software was used [23].

### Immunofluorescence staining

After 24 h treatment with PD at various concentrations or 30 μM chloroquine (autophagy positive control), cells were washed in PBS and fixed with 4% paraformaldehyde (PFA) solution and permeabilized with 0.1% TRITON -X/PBS solution, then was performed a blocking in 1% BSA for 1 hat RT. Cells were incubated with LC3B antibody (Cell Signalling, USA) and Anti-SQSTM1/p62 (ab56416, Abcam, Cambridge, UK) in PBS for 30 min. Secondary antibodies were added after a PBS wash in the same conditions. Cells were incubated in a 1:500 solution of 10 mg/mL Hoechst (Invitrogen) in PBS for 10 min in the dark. To stain endoplasmic reticulum cells were incubated with 200 nM ER-Tracker Blue-White DPX in PBS solution for 20 min at 37 °C. For positive control cells were exposed for 16 h to 5 μg/mL tunicamycin. Images were collected under a fluorescence microscope (EVOS FL Cell Imaging System, Thermo Scientific, Rockford, USA). To stain lysosomes, cells were incubated with 60 nM LysoTracker (Thermo Fisher Scientific, USA) for 45 min at 37 °C. ImageJ (Fiji plugin) software was used for the calculation of Puncta and Intensity correlation quotient (ICQ).

### FACS analysis

For intracellular staining cells were fixed with Fix and Perm Reagent A (Invitrogen) for 20 min, and then resuspended in Fix and Perm Reagent B (Invitrogen) for 30 min containing primary LC3B antibody (Cell Signalling, USA).

Secondary antibodies were added in Fix and Perm Reagent B (Invitrogen) in the same conditions. Apoptosis (Annexin V apoptosis detection kit, BD biosciences), CellROX assay (Thermo Fisher Scientific, USA), LysoTracker assay (Thermo Fisher Scientific, USA), were performed according to the manufacturer’s instructions. Cells were analyzed with a FACSAria III (BD Biosciences, San Jose, CA) or a BD Accuri Cytometer (BD Biosciences, San Jose, CA). Data were analysed by FlowJo V10 software (FlowJo LLC, USA).

### G6PD overexpression

p3-G6PD-t1 and negative control pCMV3-untagged-NCV (control) hygromycin-resistant plasmids were purchased from Sino Biological Inc. (Sino Biological, Beijing, China). MCF7 cells were stably transfected with Lipofectamine 3000 (Thermo Fisher Scientific, Waltham, MA USA) according to the manufacturer’s instructions. Clones with upregulated expression of G6PD were selected with 100 μg/mL Hygromycin. Clones were screened by Western blot.

### Protein extraction and Western blotting

Cells were lysed in 1x RIPA buffer (150 mM NaCl, 1% NP-40, 0.5% sodium deoxycholate, 0.1% SDS, 50 mM Tris. Cl pH 7.5) plus 1% protease inhibitor cocktail, 1% PMSF (200 mM) and 1% sodium orthovanadate (Santa Cruz Biotechnology, USA). Lysates were clarified by centrifugation at 8000 x g for 5 min at 4 °C and equal amounts of protein were fractionated by SDS-PAGE and subsequently transferred onto nitrocellulose membrane, immunoblots were visualized using Supersignal® West Pico Chemiluminescent substrate (Thermo Scientific, Rockford, USA). Proteins were detected with anti-Glucose 6 Phosphate Dehydrogenase (Novus Biologicals, USA); Anti-SQSTM1 / p62 (ab56416, Abcam, Cambridge, UK); anti-Phospho-Akt (Ser473) (9271, Cell Signalling, USA), anti-Akt (9272, Cell Signalling, USA); anti-phospho-mTOR (Ser2448) (2971, Cell Signalling, USA); anti-mTOR (2972, Cell Signalling, USA); anti-LC3B (2775, Cell Signalling, USA); anti-α-Tubulin Antibody (#2144 Cell Signalling Technology, UK); Anti-GAPDH (ab9485, Abcam, Cambridge, UK) were used for assessing loading.

### In silico analysis

In silico validation was performed using gene expression profiles generated as part of the Molecular Taxonomy of Breast Cancer International Consortium (METABRIC) [24] and Pawitan [25] studies. The gene expression profiles were generated using an Illumina HT-12 v3.0 Gene Expression BeadChip (METABRIC) and an Affymetrix Human Genome U133A/U133B (Pawitan) array. The gene expression of G6PD was selected and min-max normalised across the complete patient population of both datasets. The normalised gene expression was analysed using scatter plots for the differentiation of G6PD expression across breast cancer subtypes (Her2+ enriched, Basal-like, Luminal A, Luminal B and Normal-like) and disease recurrence. Unpaired t-test was used to analyse differences between sample groups. In addition, Kaplan-Meier curves were generated presenting disease-free survival (DFS) in relation to G6PD expression. Patient groups were separated according to median expression and survival curves were analysed using Mantel-Cox test. *P*-values below 0.05 were considered to be significant (*p* ≤ 0.05 = *, *p* ≤ 0.01 = **, *p* ≤ 0.001 = *** and *p* ≤ 0.0001 = ****). Analysis was performed using GraphPad Prism 8.

## Results

### G6PD inhibition induces autophagy in breast cancer cells

**Fig. 1:**
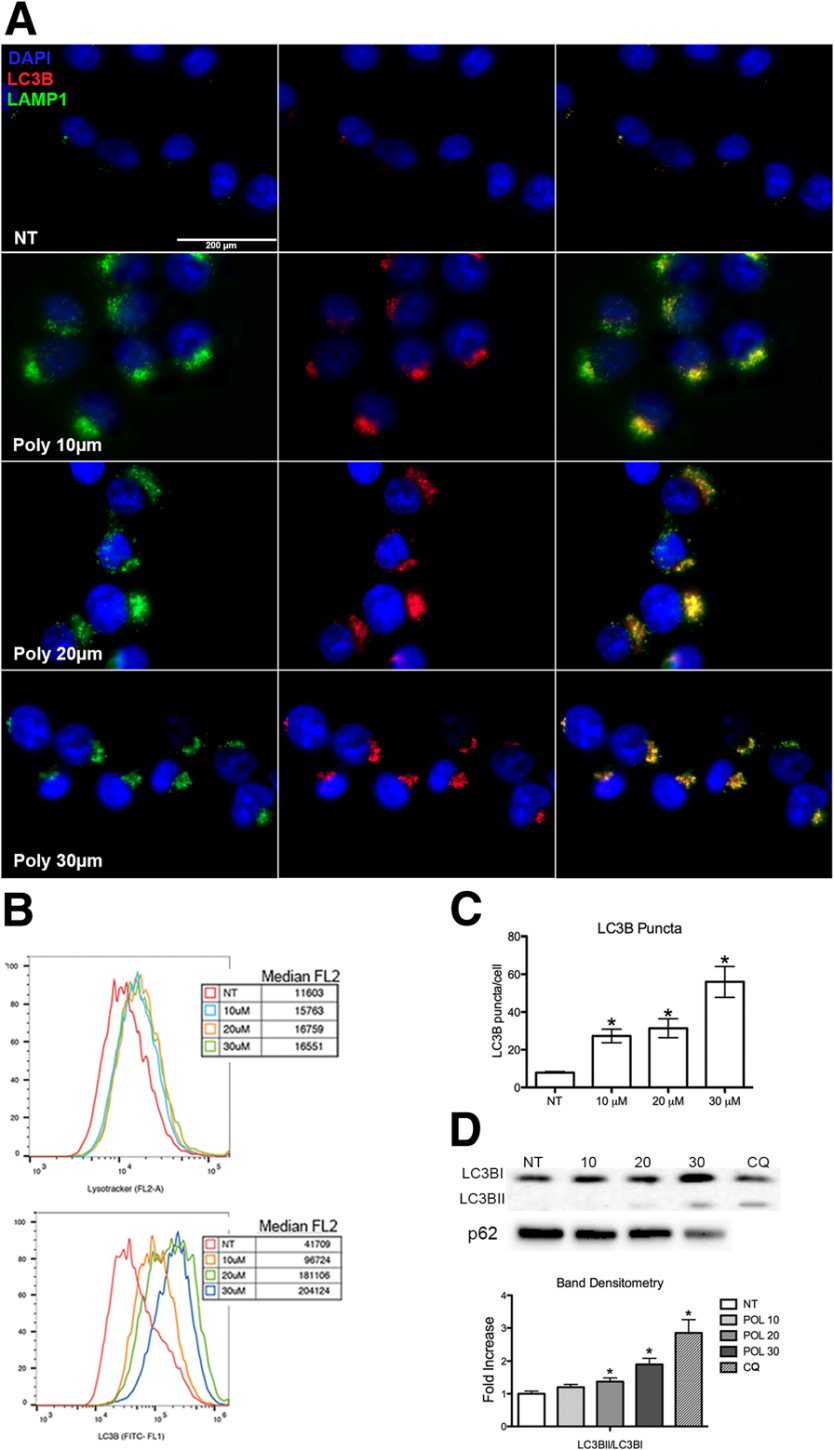
G6PD inhibition induces autophagy. **a** Autophagy analysis on polydatin-treated cells (10–30 μM) performed by immunofluorescence for LAMP1 (top row) and LC3B protein (lower row), 24 h after treatment. **b** Flow cytometry histograms for the Lysotraker and Lc3B, median fluorescence values are shown in the graphs. Both IF and flow cytometry show an increase of LAMP1, LysoTracker and LC3B fluorescence accordingly to polydatin concentration. **c** Analysis of puncta on IF for LC3B in polydatin-treated cells (10–30 μM), 24 h after treatment. Puncta represent single autophagosomes. **d** Immunoblot for LC3B and p62; band densitometry in polydatin treated cells (10–30 μM), 24 h after treatment. LC3BII increases accordingly to polydatin concentration while p62 decreases. *p* < 0.05, *N* = 3 (Biological replicates); error bars = SEM

Autophagy is a biological survival mechanism that is activated in cells during stress. Activation of this pathway in cancer cells can favour or hinder cancer progression depending on cell context. In a previous work [26], we showed that the natural molecule polydatin directly inhibits G6PD by inducing reactive oxygen species (ROS) and increasing Endoplasmic Reticulum (ER) stress. Both biological effects are frequently associated with increased autophagic flux. This led us to investigate the effect of G6PD blockade on autophagy and its role in breast cancer cells response to treatment. Macroautophagy (from now on autophagy) is a cellular process that allow for orderly degradation recycling of cellular organelles. The major steps of this process include the formation of autophagosome, vesicles containing the organelles to be degraded, and successively the fusion of these with lysosome (autophagolysosomes). Autophagosomes formation involves the recruitment of LC3B (Microtubule-associated protein 1A/1B-light chain 3) and p62/sequestosome proteins. In order to monitor autophagy in treated cells, we performed an IF and a quantitative analysis by flow cytometry using the vital dye LysoTracker, LAMP1 and LC3B antibody. LysoTraker is a fluorescent dye for labelling and tracking acidic organelles in live cells, thus it will stain both lysosomes and autophagolysosomes. Both qualitative and quantitative analyses showed a consistent increase in autophagy following exposure to polydatin (Fig. 1a,b). Lysotracker staining showed a strong increase in acidic vesicles after treatment as assessed by measuring median fluorescence ranging from 11,603 (untreated (NT)) to 16,551 (treated with 30 μM Polydatin). LC3B staining indicated the appearance of several fluorescent vesicles with a median fluorescence ranging between 41,709 (untreated) to 204,124 (treated with 30 μM Polydatin). To investigate the formation of autophagic vesicles, we analysed the LC3B puncta per cells (Fig. 1c). Typically, LC3B associated to autophagosomes is visualized in immunofluorescence as dots (puncta), each dot representing an autophagosome. We found a consistent increase of puncta in a Polydatin concentration-dependent manner. When is recruited on autophagosomes LC3B becomes conjugated with phosphatidylethanolamine (LC3BII) [27]. The activation of LC3BI (LC3BII) was also confirmed by immunoblotting (Fig. 1d) and band densitometry showed a significant increase in LC3BII following increasing Polydatin concentrations.

**Fig. 2:**
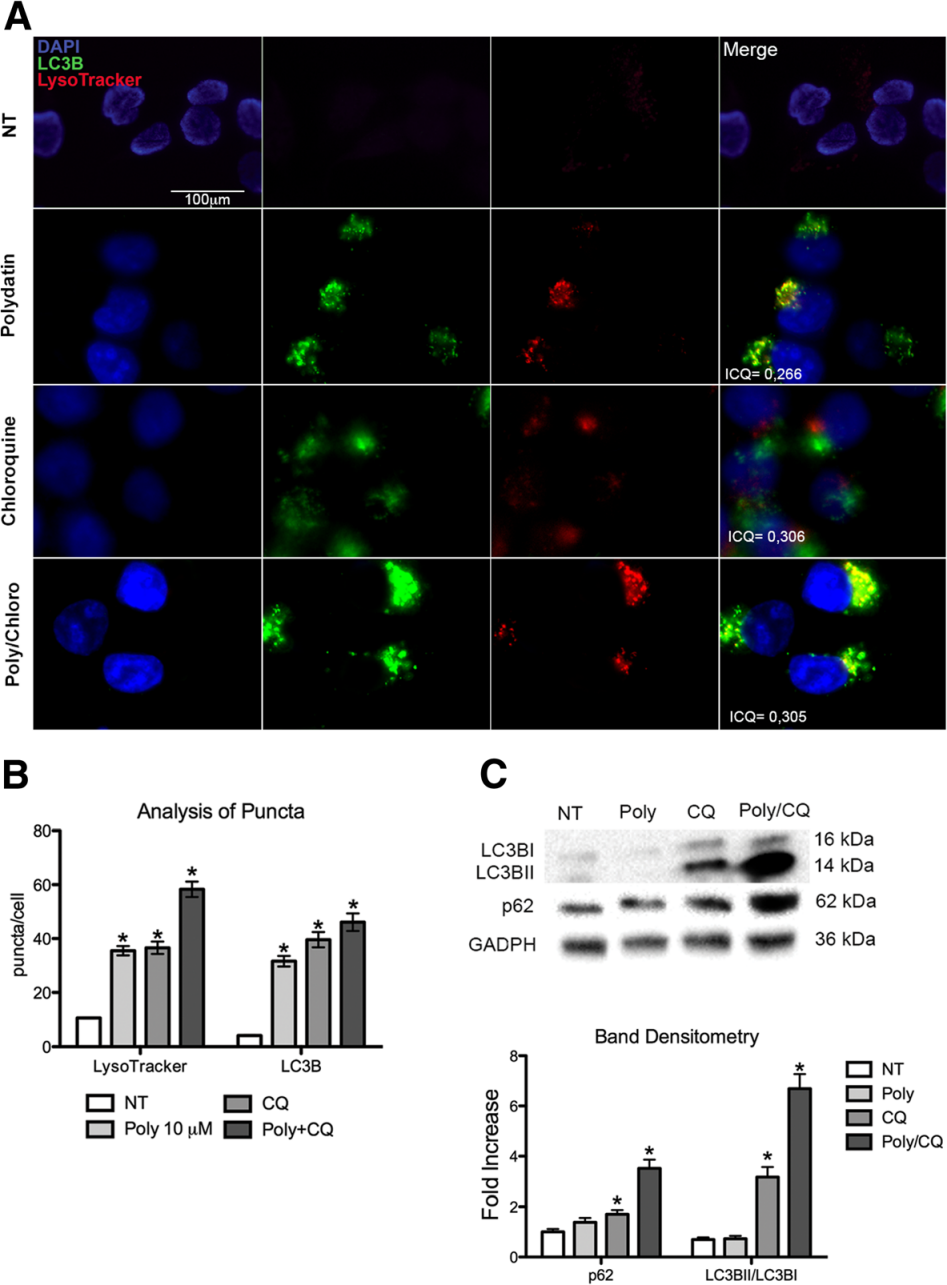
Analysis of autophagy flux. **a** IF with Lysotracker and LC3B for autophagy flux analysis on cells treated with polydatin (10 μM), chloroquine (30 μM) and their combination. Co-treatment with chloroquine increases the fluorescence of both LysoTracker and LC3B suggesting that polydatin triggers autophagy. Intensity correlation quotient (ICQ) shows the grade of co-localization of LC3B and Lysotracker; in all three treatments a colocalization grade of about 70% was recorded. **b** Analysis of puncta for IF of LC3B and Lysotracker on cells treated with polydatin (10 μM), chloroquine (30 μM) and their combination. **c** Immunoblot with band densitometry for LC3B and P62/sequestosome on cells treated with polydatin (10 μM), chloroquine (30 μM) and their combination. Increase of LC3B and p62 bands in cells co-treated with polydatin and chloroquine confirms that polydatin triggers autophagic flux. *p < 0.05, *N* = 3; error bar = 95% confidence

To better understand the effect of G6PD blockade on autophagy (e. g. if it increases or blocks autophagic flux with accumulation of autophagosomes), we performed an analysis of autophagic flux by co-treating the cells with chloroquine, a drug that block the fusion of autophagic vesicles and lysosomes, resulting in the blockade of autophagic flux and accumulation of autophagosomes. Thus, when a drug works by blocking the autophagy flux, its effect on autophagy markers (LC3B and p62) will be reduced or abrogated by co-treating with chloroquine; on the other hand, if a drug increases the autophagic flux its effect on autophagy markers will be increased. Indeed, cells treated with chloroquine showed a strong staining of both LysoTracker and LC3B by IF (Fig. 2 a, b) and increased protein expression of LC3BII and p62/sequestosome (Fig. 2c). When cells were co-treated with chloroquine and polydatin, a significant increase of LysoTracker staining and expression of LC3BII and p62/sequestosome were also observed (Fig. 2 a, b, c). Moreover, we calculated the Intensity correlation quotient (ICQ) on the IF using LC3B and LysoTracker double staining. The ICQ expresses the amount of co-localization between the two staining, that in this case ranged between 50 and 60% (ICQ = 0,26-0,32). In all these experiments the co-treatment with polydatin and chloroquine constantly resulted in a stronger signal compared to single treatments. This suggests that polydatin induced an increase of autophagic flux.

### Autophagy is dependent on ER stress

**Fig. 3:**
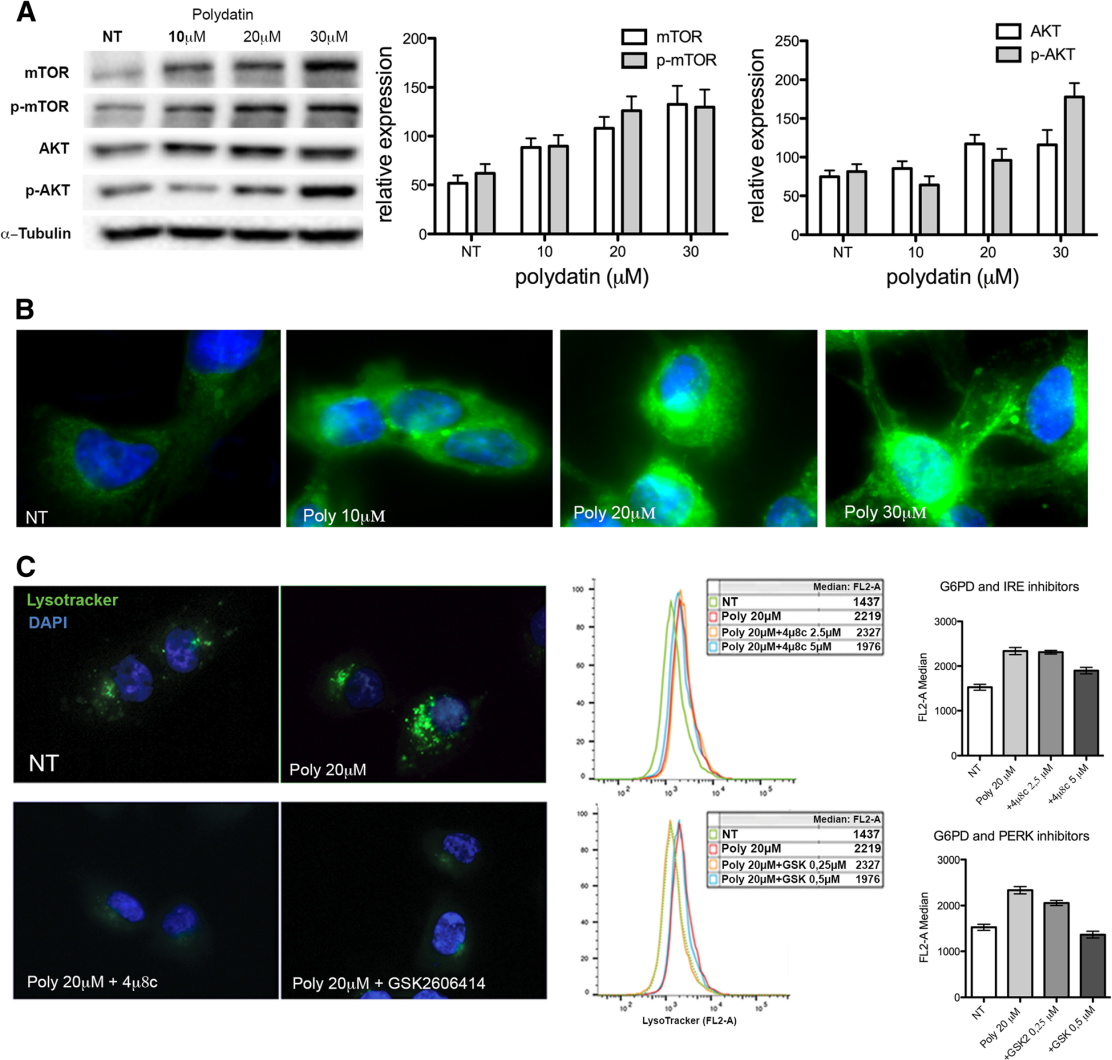
Autophagy is dependent on ER stress. **a** Immunoblot for mTOR/p-mTOR and AKT/p-AKT with band densitometries; the total amount of both AKT and mTOR increases over polydatin treatment; however, the ratio between phosphorylated and non-phosphorylated forms does not change. **b** IF with ER-Tracker 24 h after polydatin treatment. ER Tracker stains the endoplasmic reticulum and its fluorescence is proportional to ER swelling. **c** IF and flow cytometry for lysotracker on cells treated with polydatin (20 μM) and either IRE1 inhibitor 4μ8c or PERK inhibitor GSK26064141. In the flow cytometry graphs, the medians of the fluorescence intensities are plotted. Both 4μ8c and GSK26064141 reduces the effect of polydatin on autophagy. *p < 0.05, *N* = 3 (biological replicates); error bars = SEM

Autophagy can be induced by different mechanisms both in physiological and pathological conditions. One of the key pathways controlling autophagy activation is represented by the AKT/mTOR. TOR is a central regulator of cell growth and metabolism, and in response to nutritional and stress signals, it coordinates the balance between cell growth and autophagy. Polydatin has been suggested to act as an mTOR inhibitor and inducer of autophagy [28]. To exclude a potential and direct inhibitory effect of Polydatin on mTOR and to clarify its relation with G6PD inhibition, we performed immunoblots for the inactive and active (phosphorylated) forms of both AKT and mTOR (Fig. 3 a). This experiment does not show an inhibition of AKT and mTOR, on the contrary both AKT and mTOR expression increased while the ratio between the non-phosphorylated and the phosphorylated form did not change after treatments. These results suggest that the induction of autophagy was not driven by mTOR inhibition as reported for resveratrol but follows a different cell mechanism. Indeed, autophagy has been shown to be induced by UPR (Unfolded Protein Response), as a mechanism of degradation of misfolded proteins, which activation could be mediated by IRE1 or PERK [29–32]. In a previous work [26], we showed that G6PD inhibition induces a strong ER stress with activation of both PERK and IRE1. Here we confirmed a strong activation of ER stress by ER TRacker staining, this vital dye stains the endoplasmic reticulum and its fluorescence was proportional to ER swelling (Fig. 3b). Inhibition of PERK or IRE1 phosphorylation by specific inhibitors reduced autophagy in polydatin-treated cells, suggesting a causative link between ER stress and autophagy (Fig. 3c).

### G6PD overexpression reduces autophagy and induces resistance to Lapatinib

**Fig. 4:**
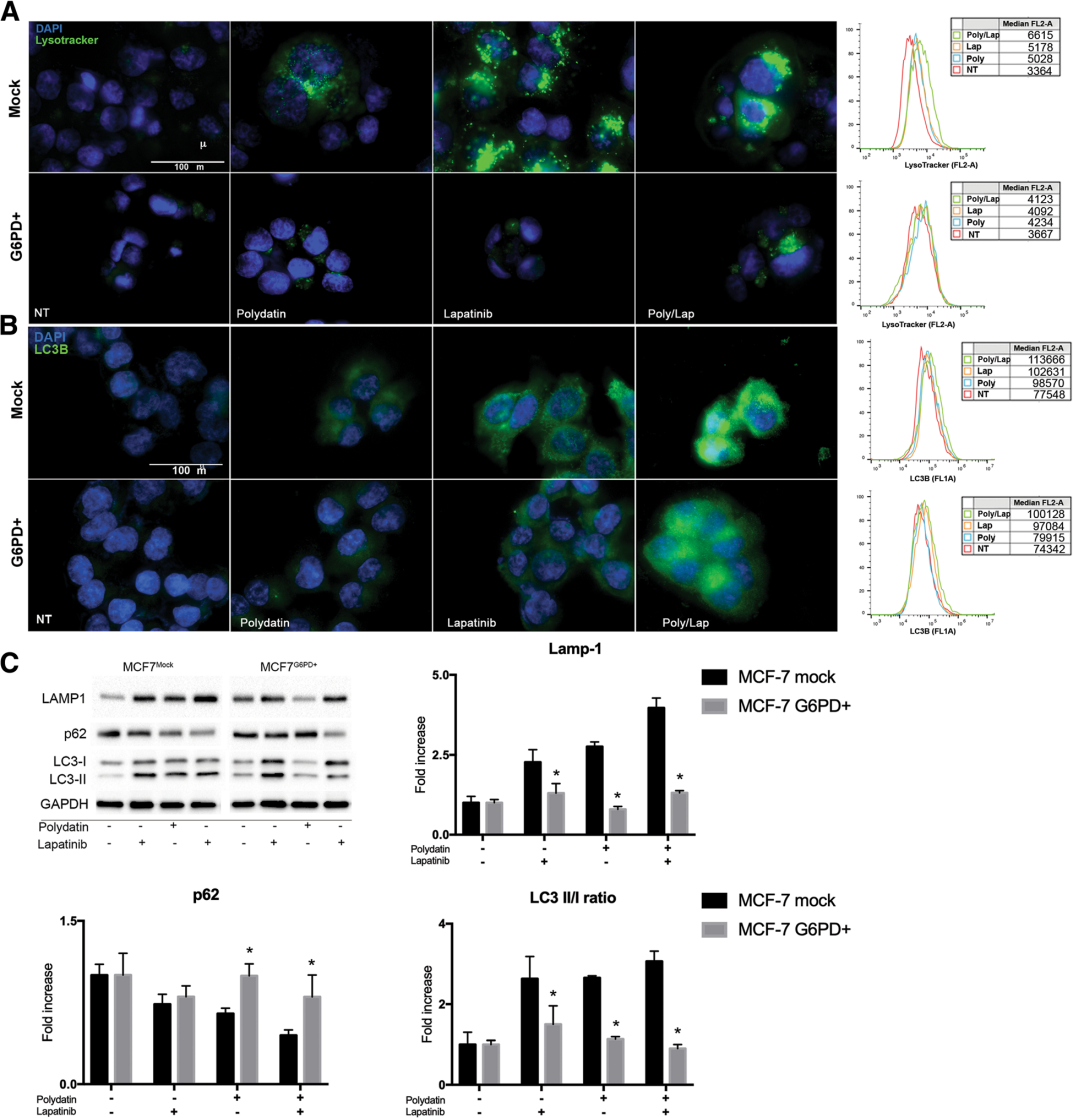
G6PD overexpression limits autophagy induction by Lapatinib. **a** IF and flow cytometry for LysoTracker on MCF7^mock^ and MCF7^G6PD+^ after polydatin (20 μM), lapatinib (20 μM) and their combination. Flow cytometry histograms show median fluorescence. G6PD+ modified cells were not responsive to both polydatin and lapatinib. **b** IF and flow cytometry for LC3B on MCF7^mock^ and MCF7^G6PD+^ after polydatin, lapatinib and their combination. Flow cytometry histograms show median fluorescence. MCF7^G6PD+^ cells were not responsive to both polydatin and lapatinib. Both IFs are example of three biological replicates that show similar results. **c** Immunoblot and band densitometry for LAMP1, p62, LC-3 on both MCF7^mock^ and MCF7^G6PD+^ treated with Lapatinib, polydatin and combinations of both drugs. *p < 0.05, *N* = 3 (biological replicates); error bars = SEM

**Fig. 5:**
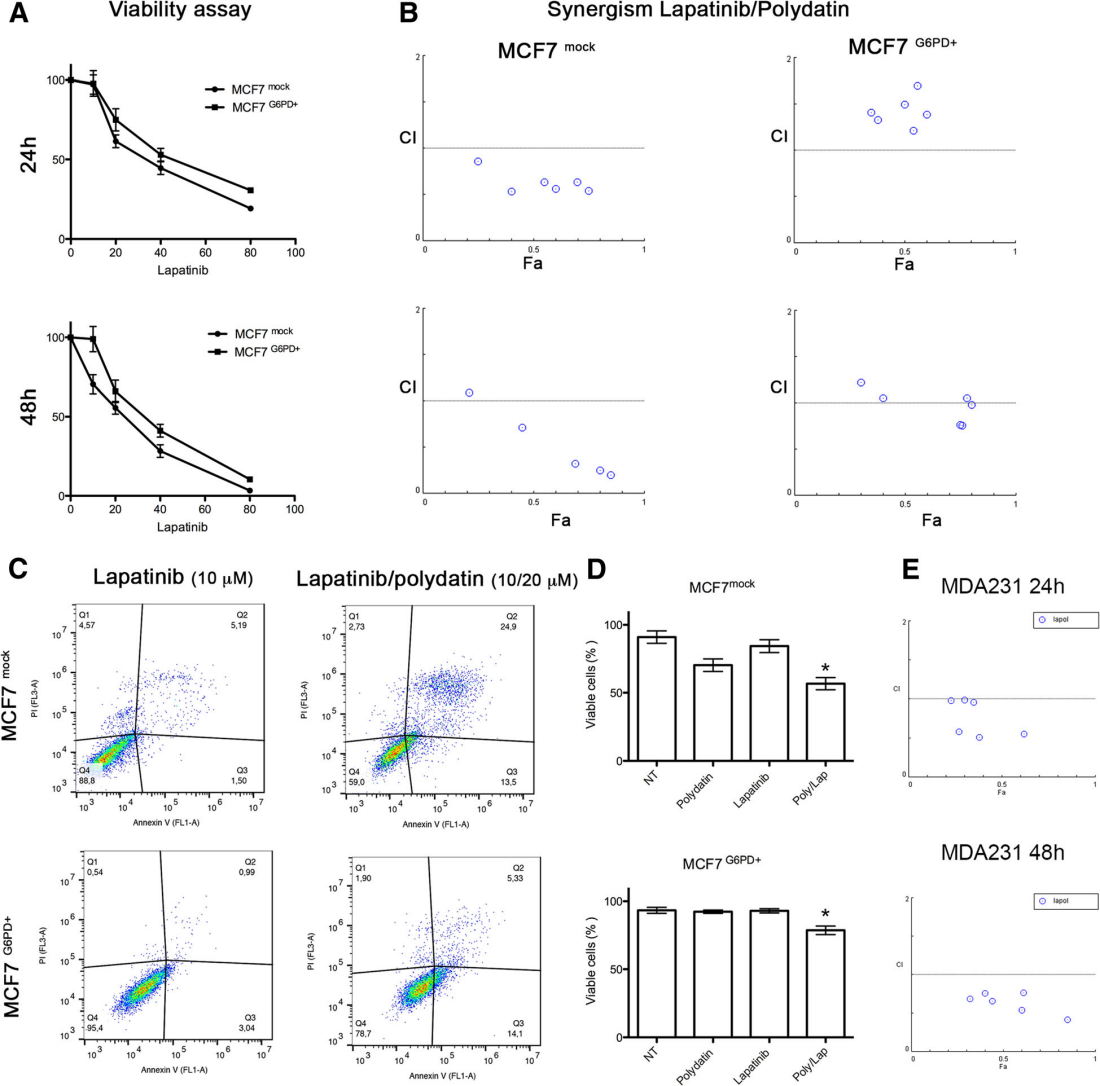
G6PD overexpression confers resistance to lapatinib while its inhibition potentiates its effect. **a** Viability assay on MCF7^mock^ and MCF7^G6PD+^ treated with Lapatinib (20 μM) at 24 h and 48 h. IC50 induced by lapatinib at 24 h was 40 μM and 51 μM, respectively, while at 48 h 19.6 μM and 37.6 μM, respectively **b** Combination index plot from Compusyn software for the analysis of the synergism induced by the combination lapatinib/polydatin. When the points are located below the threshold (1.0), it suggests that the drugs act synergistically. At both 24 and 48 h for the beginning of the treatment, polydatin and lapatinib show a significant synergism. **c** Annexin V/PI flow cytometry analysis to determine apoptosis in lapatinib and polydatin/lapatinib-treated cells. Drug combination is more effective on both MCF7^mock^ and MCF7^G6PD+^ if compared to single treatments. The effect on MCF7^mock^ is markedly stronger. **d** Histograms of the results obtained in Annexin V/PI assay shown in C. **e** Combination index plot from Compusyn software for the analysis of synergism of the combination lapatinib/polydatin on MDA-MB-231 cell line. The synergism between polydatin and lapatinib is confirmed also on this cell line. * *p* < 0,05, *N* = 3 (biological replicates); error bar = SEM

Lapatinib is an orally active drug for breast cancer therapy. It is a tyrosine kinase inhibitor which acts on both HER2/neu and epidermal growth factor receptor (EGFR) [33]. Lapatinib has been shown to induce both apoptosis and autophagy in cancer cells [19–21, 34]. Based on this, we hypothesised that G6PD blockade could increase Lapatinib effect on cancer. On the other hand, G6PD overexpression is common in several cancers and correlates with aggressiveness and poor prognosis [7, 8, 35]. Thus, we generated a cell line overexpressing G6PD (MCF7^G6PD+^) [26] and compared the activation of autophagy with control cells (mock plasmid MCF7^mock^) when treated with both polydatin and lapatinib. Lapatinib concentration was chosen from a viability assay performed on MCF7 (Fig. 5 a) to be the minimum effective concentration. As shown in Fig. 4, lapatinib induced autophagy in MCF7^mock^ but on much less extend on MCF7^G6PD+^ cells as visualized with both lysotracker (Fig. 4 a) and LC3B (Fig. 4 b) for IF and flow cytometry. Interestingly, the combination lapatinib/polydatin resulted in increased activation of autophagy. In order to confirm these data, we performed an immunoblot for LAMP1, p62 and LC-3. LAMP1 increases with all treatment on MCF7^mock^ confirming an increase in the lysosomal compartment. On MCF7^G6PD+^ polydatin didn’t produce any increase in LAMP1 while lapatinib and combo did but at much less extend than on MCF7^mock^. p62 decreased on MCF7^mock^ with all treatments and especially with the combo, on MCF7^G6PD+^ only the combo induces a decrease. LC-3BII/LC-3BI ratio.

To show if the effect on autophagy was reflected on cell viability, we performed a viability assay and studied the pharmacological synergism of the combination using Chou-Talalay method [23]. The Viability assay (Fig. 5 a) showed a significant difference between MCF7^G6PD+^ and MCF7^mock^ both at 24 h and 48 h after treatment. Interestingly, lapatinib and polydatin had a synergic effect on MCF7^mock^ but no effect was observed on MCF7^G6PD+^cells. These data were confirmed by apoptosis analysis with Annexin V/PI and assessed by flow cytometry (Fig. 5 c, d). In order to show that the synergic effect was not limited to MCF7 cells line we performed a similar experiment on MDA231 cells line obtaining similar results (Fig. 5e).

### Autophagy mediates cell death and synergism

**Fig. 6:**
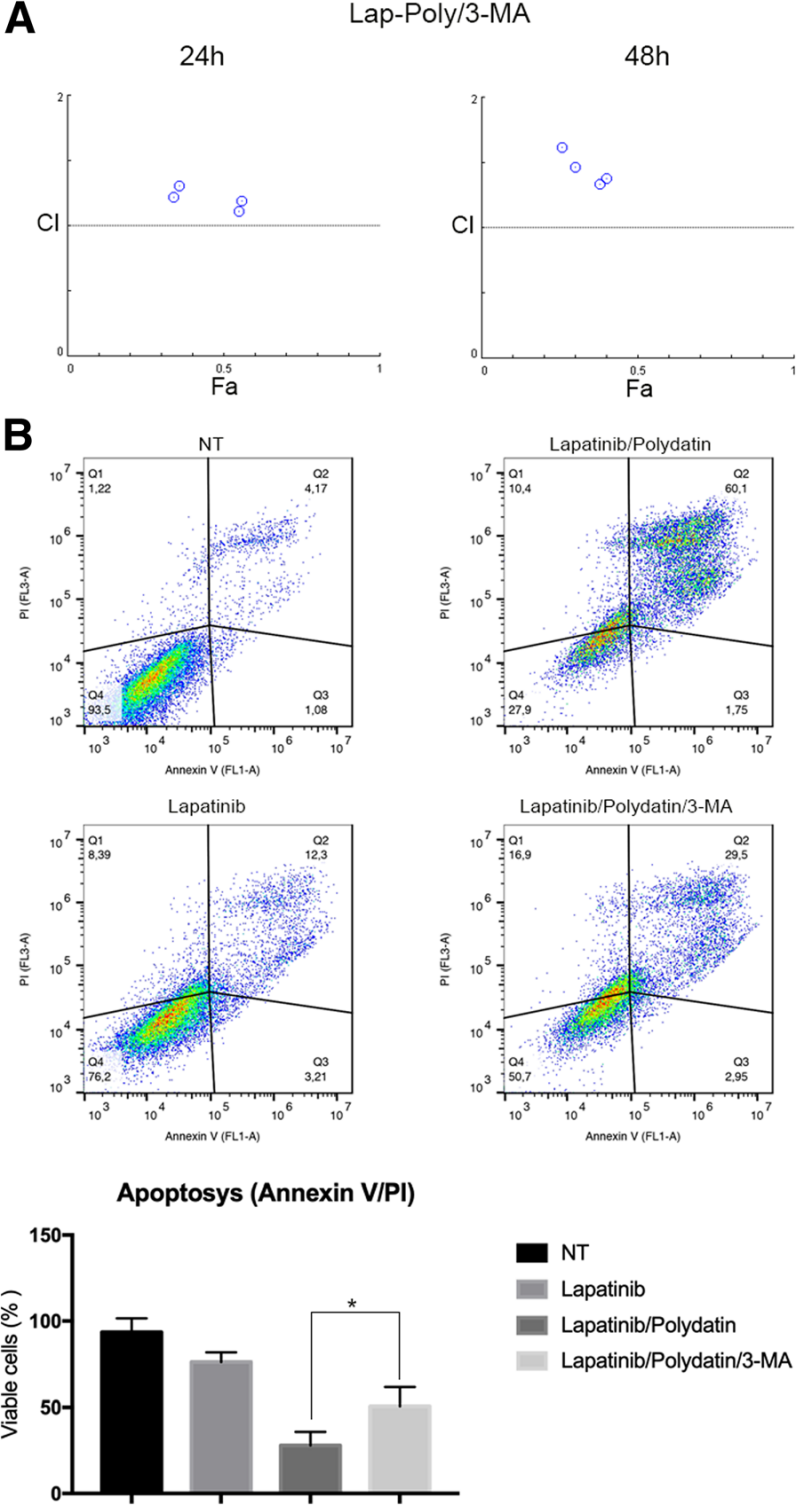
Autophagy-mediated cell death. **a** Synergism between polydatin and lapatinib in presence of 3-MA. All points are above the threshold suggesting that 3-MA counteracts polydatin/lapatinib effect and that autophagy is responsible for the synergism. **b** Annexin V/PI assay after co-treatment with polydatin/lapatinib in the presence of 3-MA. Viable cells change from 27.9% in polydatin/lapatinib-treated cells to 50.7% in presence of 3-MA. Bar graph summarizing the results of apoptosis analyses, statistical analysis show a significant difference between lapatinib/polydatin and lapatinib/polydatin/3-MA treatments. *P* < 0,05, *N* = 3; error bar = Standard deviation

To determine if autophagy was responsible for cell death and synergistic effect between polydatin and lapatinib, we performed a study of synergism in presence of the autophagy inhibitor 3-methyladenine (3-MA). This molecule blocks autophagosome formation at earlier stages by inhibiting class III PI3K [36]. 3-MA (1 MM) concentration was chosen accordingly to the literature [36–39]. When autophagy is blocked the synergic effect seen by co-treating with polydatin and lapatinib was lost (Fig. 6 a) resulting in a combination Index (CI) that was always higher than 1 (synergism is shown by a CI lower than 0.8. These data were confirmed by apoptosis analysis (Fig. 6 b). In fact, lapatinib caused an about 25% reduction of viability, its combination with polydatin induced a 75% reduction, and when 3-MA was added together with polydatin and lapatinib cell viability was reduced of only 50%. 3-MA counteracted the effect of lapatinib-polydatin combination, strongly suggesting that the cytotoxic effect obtained by inhibiting G6PD and Lapatinib was caused by increased autophagy.

### G6PD expression is correlated to disease-free survival (DFS) and recurrence in breast cancer patients

**Fig. 7:**
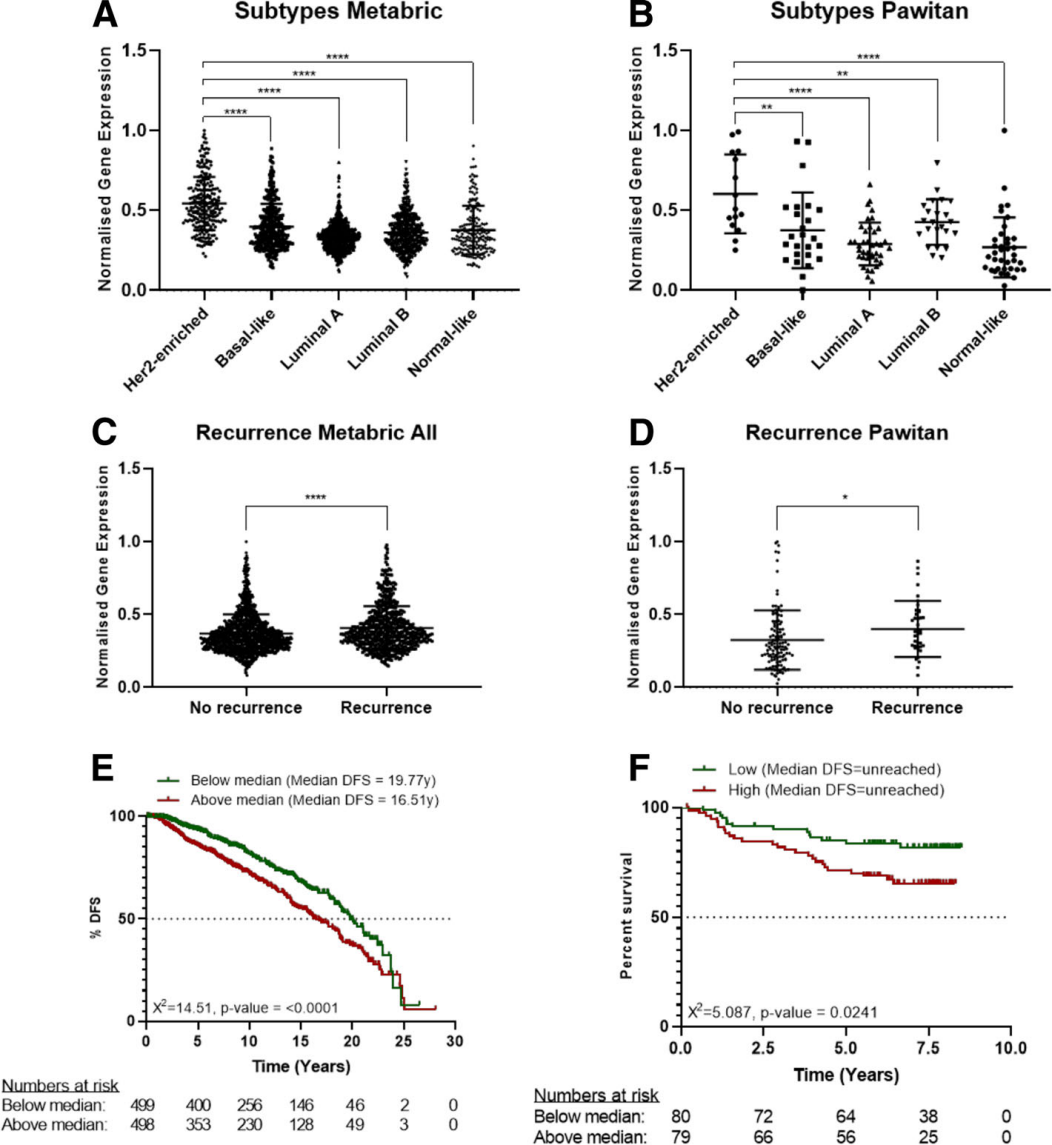
G6PD is inversely correlated to DFS in breast cancer patients. **a**, **b** Scatter plot presenting the normalized gene expression of G6PD within breast cancer subtypes. Significance was tested comparing the expression in Her2+ population with other subtypes. Results highlight that the expression of G6PD is significantly higher in the Her2+ population supporting the use of a G6PD inhibitor for this group of patients. **c**, **d** Scatter plot representing the normalized gene expression of G6PD in patients with disease-free survival (DFS) and patients with disease recurrence. Significant differences in the expression of G6PD is shown in the Metabric and Pawitan dataset. Both show a higher expression of G6PD in patients with disease recurrence compared to patients without disease recurrence. Note: The analysis was performed using all sample population based on the reduced numbers of Her2+ patients and the number of recurring and non-recurring patients within this selection. **e**, **f** Kaplan-Meier analysis on DFS after a median split. Equal number of patients were in both groups. Significant differences in DFS are shown in the Metabric and Pawitan dataset. Both show that an increased expression of G6PD results in an earlier time of relapse. Note: The difference in the graphs of Metabric and Pawitan are based on the shorter length of follow-up of Pawitan compared to Metabric. The analysis was performed using all sample population based on the reduced numbers of Her2+ patients and the number of recurring and non-recurring patients within this selection

To identify the role and impact of *G6PD* in breast cancer patients, an in silico analysis of two public available datasets generated through the analysis of patient-derived material was performed. The expression of *G6PD* across 5 breast cancer subtypes (Her2+ enriched, Basal-like, Luminal A, Luminal B and Normal-like), showed a statistically significant higher expression of *G6PD* in Her2+ enriched tumour material, compared to specimens derived from other breast cancer subtypes (Fig. 7a+b). Furthermore, the comparison of *G6PD* expression in patients with and without disease recurrence, measured at the time of diagnosis, showed a statistically significant higher expression of *G6PD* in patients with future disease-recurrence when compared to patients without future disease recurrence (Fig. 7c+d). The significance of *G6PD* on disease-recurrence was further supported through a Kaplan-Meier analysis of disease-free survival (DFS) time against median expression of *G6PD*. This analysis correlated an earlier disease-recurrence with an increased expression of *G6PD* at the time of diagnosis (Fig. 7e+f). The analysis of the METABRIC dataset has shown that patients with a lower *G6PD* expression have a 3-year longer median DFS compared to patients with a high *G6PD* expression (Fig. 7e). Similar trends were shown for the Pawitan dataset (Fig. 7f), however both groups did not cross the 50% survival mark.

## Discussion

In this manuscript, we describe for the first time that the inhibition of G6PD causes an activation of autophagic flux, which synergistically increases the cytotoxic effect of Lapatinib on breast cancer cells. Pentose phosphate pathway (PPP) is a major player in glucose catabolism that results in the production of NADPH, necessary to control redox balance, lipid metabolism and nucleotide precursors [6, 40]. G6PD is the limiting enzyme of the PPP and it is finely regulated following the cell redox state and metabolic needs. PPP activity and G6PD itself are often upregulated in cancer and are associated with aggressiveness, drug resistance and poor prognosis [7–9]. Thus, inhibition of this pathway has been pointed out as a major goal for the definition of new cancer treatments [4]. Indeed, targeting G6PD results in cancer cytotoxicity, reduction of metastases and restoration of sensitivity to drug [11, 12, 26, 35]. Nevertheless, the choice among G6PD inhibitors is very limited and debated [41]. Recently our group discovered that the natural molecule polydatin directly inhibits G6PD causing oxidative stress, endoplasmic reticulum stress and apoptosis in cancer cells [26]. Polydatin is a glucoside of resveratrol that has been studied for many years and for different pathological conditions, including inflammation and cancer [42–44]. MCF7 is most widely used breast cancer cell line for in vitro research [45]. PPP and G6PD have been proven to play an important role in their metabolism [15, 46, 47] as well as they have been used to study lapatinib effect and resistance especially in combination with other drugs [48, 49]. Here, we show that, G6PD inhibition induced an increase of both intracytoplasmic vesicles (puncta) that colocalize with Lysotracker, a marker of acidic compartments, and LC3B which is associated to autophagosomes. During autophagy induction, LC3-I is converted into LC3-II with a concurrent decrease in p62. LC3B activation was confirmed also by immunoblotting.

In the study of autophagy flux, inhibition of lysosomal degradation by chloroquine or bafilomycin A causes accumulation of both LC3-II and p62, and this reflects the amount of LC3-II and p62 that would have been degraded by autophagy over the treatment period [50–52]. In Fig. 2c the increase of p62 and LC3B in cells co-treated with chloroquine is evident. Other studies using metabolic inhibitor show an increase in autophagic flux leading to cells death [53], this corroborate our strategy to use metabolic autophagy inducer to potentiate anticancer drug effect.

mTOR is a master regulator of cell metabolism by controlling autophagy, among other processes. Typically, when mTOR is inactive or inhibited, autophagy occurs. Therefore, most of the molecules that influence autophagy acts directly or indirectly on the mTOR pathway [54]. Resveratrol, which can be produced from polydatin degradation, has been described as mTOR inhibitor [28, 55, 56]; thus, to exclude a direct effect of polydatin on this pathway, we analysed the activation of mTOR and its upstream regulator AKT. We showed that both AKT and mTOR are not inhibited. A possible case in which autophagy is induced independently from mTOR is represented by the unfolded protein response process that is initiated following ER stress [57, 58]. Indeed, both IRE1 and PERK, which are the principal mediator of UPR, have been described to induce autophagy independently from mTOR status [39, 59]. Our results confirmed [26] that G6PD inhibition caused ER stress and that, if UPR was inhibited by blocking IRE1 or PERK, polydatin was not still able to induce autophagy.

Tyrosine kinase inhibitors are a class of drugs that are largely used in clinical settings to treat different cancers including breast carcinoma. The work of Gregory et al. [22] pointed out that G6PD is responsible for resistance to FMS-like tyrosine kinase 3 inhibitors in acute myeloid leukaemia due to increased redox metabolism. Among TKIs that are used to treat breast cancer, lapatinib has been described to cause cancer cells cytotoxicity by inducing autophagy [19, 34]. Therefore, we hypothesized that G6PD might play a role in modulating lapatinib effect on cancer cells. For this purpose, we created a cell line overexpressing G6PD and showed that they are resistant to autophagy induced by either lapatinib or polydatin. On the other hand, cells that received the mock plasmid showed a strong induction of autophagy, especially with the combination of the two drugs. These results were confirmed by viability analyses where the MCF7^G6PD+^ were less sensitive to lapatinib. Moreover, the analysis of synergism between polydatin and lapatinib showed that the two molecules were highly synergistic in the MCF7^mock^ at both 24 h and 48 h after treatment, while this effect was less evident or absent on the MCF7^G6PD+^. These results were confirmed by Annexin V/PI analysis. Moreover, to confirm the role of autophagy in cancer cell death, we analysed both synergism and apoptosis in the presence of 3-metyladenin (3-MA), which is a widely used autophagy inhibitor [37, 60]. Differently from chloroquine and bafilomycin A, 3-MA inhibits autophagosomes formation at early stages. For these reasons, 3-MA has been widely used in similar experiments [38, 60]. In these experimental conditions, synergism was completely prevented and cell viability increased from about 27% in polydatin/lapatinib-treated cells to about 50% in the presence of 3-MA. In silico studies on two publicly available patient’s databases showed a significant association between G6PD and HER2+ patients and an inverse correlation between G6PD expression and DFS. These data were on line with other reports that used different databases [7, 8]. Possible explanation of the synergistic mechanism showed here involve autophagic cell death. Shimuzu et al. showed that Bcl-2 family of protein, which is a regulator of apoptosis, control autophagic cell death by binding to Beclin-1 and APG5 [61]. Moreover, Ros induce JNK phosphorylation can activate autophagy by interfering with the interaction between Beclin-1 and bcl-2 [62]. In addition, another mechanism that could be involved in this process is a novel programmed autophagic cells death depend on iron called ferroptosis. This involved an increase of lipid peroxides and excessive degradation of ferritin and NCOA4 [62, 63]. Future studies are necessary to understand the precise role of this mechanisms and pathways in the context described in this manuscript.

## Conclusion

Taken together, our results led to the following conclusions: i. G6PD blockade caused autophagy through ER stress; ii. G6PD activity influenced lapatinib effect on cancer cells by preventing autophagy; iii. G6PD inhibitors such as polydatin might be used to increase lapatinib effect on breast cancer.

Cancer is more and more evidenced as a complex disease in which metabolic pathways play a fundamental role, either for the growth, metastases or resistance to treatments. It becomes evident that targeting these pathways is necessary to develop effective and resolute strategies. Here we provide an example in which the co-targeting of PPP and tyrosine kinase receptors lead to synergistic effects on breast cancer cells. This approach could be easily translated into clinical setting.

## Abbreviations

3-MA: 3-methyladenine
CI: Combination Index
DFS: Disease-free survival
EGFR: Epidermal growth factor receptor
ER: Endoplasmic Reticulum
ERec: Oestrogen receptor
G6PD: Glucose-6-phsphate dehydrogenase
HER-2: Human epidermal growth factor receptor 2
ICQ: Intensity correlation quotient
IF: Immunofluorescence
IRE1: Inositol-requiring enzyme 1
LAMP1: Lysosomal-associated membrane protein 1
LC3B: Microtubule-associated proteins 1A/1B light chain 3B
NADP+: Nicotinamide adenine dinucleotide phosphate
PERK: Protein kinase R (PKR)-like endoplasmic reticulum kinase
PPP: Pentose phosphate pathway
PR: Progesterone receptor
ROS: Reactive oxygen species
TKI: Tyrosine kinase inhibitor
UPR: Unfolded Protein Response
WB: Western Blot
